# A stand-alone lateral condyle-elevating trochlear osteotomy leads to high residual instability but no excessive increase in patellofemoral osteoarthritis at 12-year follow-up

**DOI:** 10.1007/s00167-017-4602-y

**Published:** 2017-06-16

**Authors:** S. Tigchelaar, J. van Sambeeck, S. Koeter, A. van Kampen

**Affiliations:** 1 Department of Orthopaedic Surgery, Radboud University Medical Center, Nijmegen, The Netherlands; 2 Department of Orthopaedic Surgery, Canisius-Wilhelmina Ziekenhuis, Postbus 9015, 6500 GS Nijmegen, The Netherlands

**Keywords:** Trochlear dysplasia, Trochleoplasty, Trochlear osteotomy, Patellar instability, Osteoarthritis

## Abstract

**Purpose and hypothesis:**

Trochlear osteotomy is a rarely performed procedure, only indicated in selected cases. Due to its nature, it can potentially lead to cartilage damage and subsequent early osteoarthritis. Satisfactory short-term results from lateral condyle-elevating osteotomy have previously been reported. The long-term effects of this procedure on clinical outcomes, patellar stability and radiological osteoarthritis are reported here.

**Methods:**

Sixteen patients (19 knees) with patellar instability due to trochlear dysplasia were included. An isolated lateral condyle-elevating trochlear osteotomy was performed between 1995 and 2002. All patients were re-examined at a minimum of 12-year follow-up. Three patients were lost to follow-up, and one patient underwent a patellofemoral arthroplasty 3 years post-operatively due to progressive osteoarthritis. Complete follow-up was therefore available in 12 patients (15 knees). Recurrent instability, VAS pain, WOMAC, Lysholm and Kujala scores were used as outcome measures. Radiological osteoarthritis was recorded using the Iwano and the Kellgren–Lawrence classifications. A repeated-measures ANOVA was used to test for repeated measures (pre-operative, 2-year and final follow-up), and Spearman’s correlation coefficient for relationships between osteoarthritis and functional scores.

**Results:**

At final follow-up, VAS pain showed a non-significant improvement from 52 to 25, and the median Kujala score was 78. Median Lysholm (54–71, *p* = 0.021) and WOMAC (78–96, *p* = 0.021) scores improved from the pre-operative assessment to final follow-up. There was no significant difference observed between clinical scores at the 2-year and final follow-up. Residual patellar instability was reported in four out of 15 knees. Three knees showed no patellofemoral osteoarthritis, eight knees had grade 1 and four knees grade 2. No correlation between VAS pain, Lysholm, WOMAC or Kujala scores and osteoarthritis could be identified (n.s.).

**Conclusion:**

A stand-alone lateral condyle-elevating trochleoplasty results in the significant improvement of most clinical scores; however, when performed as a stand-alone procedure, it leads to a high percentage of residual instability. In contrast to general belief, the development of patellofemoral osteoarthritis at 12-year follow-up did not exceed the findings from other trochleoplasty case series.

**Level of evidence:**

Case series with no comparison group, Level IV.

## Introduction

Patellar instability is associated with a number of predisposing factors [8], with trochlear dysplasia as the largest contributor preventing lateral patellar displacement [25, 26]. In patients with persistent patellofemoral instability due to trochlear dysplasia, a trochleoplasty can be an effective way to permanently regain stability [4, 31], especially in cases with high-grade trochlear dysplasia or when other procedures have failed [5]. Based on the Dejour classification [7], trochleoplasty may be indicated for trochlear dysplasia grade B, C and D [5, 19, 21].

The main goal of trochleoplasty is to restore the lateral bony restraint to normal biomechanical parameters. Due to the nature of this procedure, potentially complications include cartilage damage and an increased risk of early osteoarthritis, as the congruency of the articulating surface of the distal femur is altered [21]. Multiple techniques and specific procedures for trochleoplasty have been described [1, 5, 11, 21, 33]. In contrast to sulcus-deepening trochleoplasty, a procedure in which the central part of the trochlea is deepened [21], another option is to raise the lateral side of the trochlea through an elevating anterior lateral femoral condyle osteotomy. A primitive version of this technique was described by Albee [1]. Although most review articles mention this technique as a reasonable option [19], literature regarding the results of this technique is limited [3, 34]. The only two previous reports are of poor methodologic quality and include a limited number of patients. The general perception is that a lateral condyle-elevating trochleoplasty will raise patellofemoral contact pressures and therefore initiate patellofemoral pain and cartilage degeneration [21]. The scientific evidence for this presumption is scarce, only being supported by one biomechanical study by Kuroda et al. [18], who found raised pressures when the trochlea was elevated 6–10 mm in a cadaver model. We have used this procedure for high-grade trochlear dysplasia and in patients with trochlear dysplasia in which other procedures failed. We have previously published short-term results of this form of trochlear osteotomy in these difficult to treat patients [16]. No residual instability was seen in 17 of 19 knees, and marked improvement in pain and functional scores was found in most patients at a mean follow-up of 51 months; results were regarded as satisfactory. In order to establish the long-term results of this procedure, these same patients were re-evaluated to investigate the long-term outcomes of this lateral condyle-elevating trochlear osteotomy. This study aims to describe the clinical and radiological results of the largest series of patients to date with this surgical technique at a minimum of 12 years post-operatively with emphasis on (1) the clinical results with regard to functional scores and patellar stability and (2) the occurrence of osteoarthritis. It was hypothesized that both the clinical and radiological results would deteriorate over time.

## Materials and methods

In the previous study, the results from 16 consecutive patients (19 knees) were reported [16]. Inclusion criteria were objective patellar instability due to isolated trochlear dysplasia as established on true lateral conventional radiographs, and closed physes. Patients were treated with a stand-alone anterior lateral femoral osteotomy without simultaneous procedures between 1995 and 2002 by the senior author (AK; see “Operative technique” section). All 16 patients (19 knees) were approached to participate in this study. Two patients were unwilling to participate, and one patient could not be contacted and was considered lost to follow-up. As reported in our previous study [16], one patient had a patellofemoral arthroplasty 3 years after her trochlear osteotomy, at age 46 years, due to progressive osteoarthritis. Grade 2 osteoarthritis on the Iwano scale was seen at time of the trochlear osteotomy and grade 3 when she received the patellofemoral arthroplasty. She underwent a lateral release and medial reefing prior to her trochlear osteotomy. Due to the nature and inherent drawbacks of this procedure, we were unable to compare her results (clinical, radiological and functional scores) with the other patients. Follow-up could therefore be obtained in 12 patients and 15 knees. Fourteen knees were available for physical evaluation, as one patient was unwilling to travel to the clinic. She did, however, complete the scores, and radiographs were performed elsewhere. Data collection was conducted at a single outpatient visit. Medical history since the most recent follow-up and validated questionnaires were obtained including late complications, subsequent surgeries, the number of recurrent dislocations, VAS pain and Lysholm [28] and WOMAC knee scores. The Kujala [17] score was not previously recorded, but was noted at final follow-up to allow comparison of our study results with those of other studies.

Physical examination of the knee was performed. In patients who had undergone bilateral surgery, each knee was evaluated separately. The function and stability of the knee were examined using tests for maltracking and instability, including the apprehension test and the Rabot and J-signs. Conventional radiographs were taken in an AP, true lateral [15] and 30° patella skyline view. Osteoarthritis was graded using the Kellgren–Lawrence (K–L) scale [14] in the medial and lateral compartments of the tibiofemoral joint and using the Iwano scale [12] for the patellofemoral joint. The median follow-up was 13.6 years (range 11.9–19.0 years). Median age at follow-up was 38.1 years (range 28.9–47.1 years). Three males (four knees) and nine females (11 knees) participated. Seven knees had undergone realignment procedures of different kinds prior to trochlear osteotomy. These included two Roux–Goldthwait procedures, two tibial tubercle transfers, one medial reefing and lateral release, one combined tibial tubercle transfer and medial reefing, and one knee was treated with a medial reefing, lateral release and subsequently a varus-inducing osteotomy. An arthroscopy alone was performed in one knee, and seven knees had not undergone any previous surgeries. None of the patients had undergone a previous MPFL reconstruction, as it was not an often-performed procedure at that time.

After trochlear osteotomy, one patient underwent multiple surgeries (including an MPFL reconstruction), one patient had a tibial tubercle distalisation, and one patient had a tibial tubercle distalisation and medialisation 2 months post-operatively (all patients unilateral), due to persistent instability. None of the patients who underwent additional procedures post-trochlear osteotomy had subsequent persisting instability. Twelve knees (nine patients) did not undergo any further surgeries.

Where possible, results were compared to the pre-operative values and the results at the 2-year follow-up.

### Operative technique [16]

**Fig. 1:**
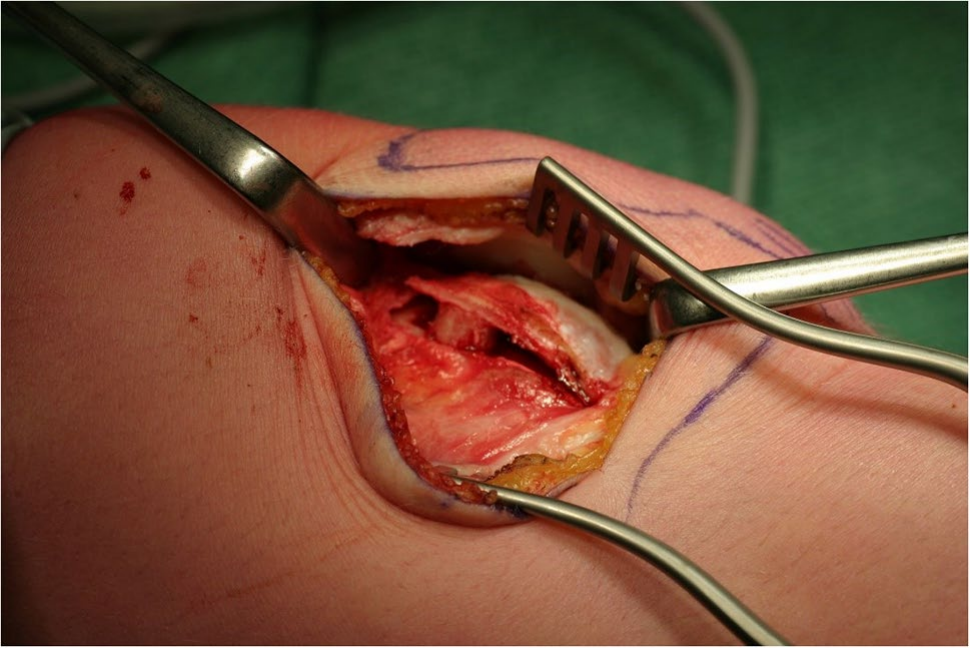
Lateral condyle-elevating trochlear osteotomy. The osteotomy extends from the beginning of the trochlea proximal and extends to the sulcus terminalis distal. It is secured by a bonegraft from the iliac crest

After skin incision, a lateral parapatellar incision was made and extended distally along the lateral femoral condyle. To visualize the osteotomy, two Kirschner wires were placed in the direction of the osteotomy until visible through the cartilage (halfway between the medial and lateral femoral facet). A curved incomplete osteotomy with small osteotomes from the beginning of the trochlea proximally to the subchondral bone of the sulcus terminalis distally was performed. The lateral articular surface of the trochlea was levered 6–8 mm, and the osteotomy was secured with a wedge-shaped autograft taken from the ipsilateral iliac crest (Fig. 1). Fixation of the osteotomy with osteosynthesis material was not necessary. The synovium was closed over the previously performed osteotomy; the lateral retinaculum was left open (i.e. a lateral release). This was done in order to avoid over-tightening the lateral structures after the lateral condyle was raised. Post-operatively, patients were placed on a continuous passive motion device (CPM) to stimulate a passive range of motion until knee flexion was at least 60°. Patients were advised partial weight bearing without flexion limitation for the first 6 weeks post-operatively.

### Ethical approval

The study received ethical approval from the regional institutional review board (IRB) Arnhem-Nijmegen (IRB registration number: NL48316.091.14), and all participants provided informed consent.

### Statistical analysis

Data analysis was performed using SPSS version 20.0 (IBM SPSS Statistics for Windows, IBM Corp., Armonk, NY, USA). A repeated-measures ANOVA was performed to test for repeated measures (pre-operative, 2-year and final follow-up) of VAS pain, Lysholm and WOMAC scores. Correction for multiple comparisons was done using the Fisher’s least significant difference test. Spearman’s correlation coefficient was calculated between the grades of osteoarthritis of the patellofemoral joint and the medial and lateral tibiofemoral joint and the VAS pain, WOMAC, Kujala and Lysholm scores. For all datasets, differences with *p* values <0.05 were considered statistically significant.

## Results

The median values for VAS pain, Kujala, Lysholm and WOMAC scores pre-operatively, at 2-year and at final follow-up are depicted in Table 1. All scores showed an improvement from pre-operative to 2-year follow-up, and no significant deterioration was seen from 2-year to final follow-up.

**Table 1:** Median values for VAS pain, Kujala, Lysholm and WOMAC scores pre-operative, at 2-year follow-up and at final follow-up

Score	Pre-operative	2-year follow-up	Final follow-up	*p* value (figures in bold are significant)
VAS pain (range)	52.0 (7–95)	15.0 (0–60)	25.0 (0–66)	* ***p*** **=** **0.003** ^†^(n.s.) ^ǂ^(n.s.)
Lysholm	54.0 (27–78)	87.0 (54–100)	71.0 (35–100)	* ***p*** **=** **0.000** ^**†**^ ***p*** **=** **0.021** ^ǂ^(n.s.)
WOMAC	78.0 (50–100)	93.5 (65–100)	95.83 (74–100)	* ***p*** **=** **0.012** ^**†**^ ***p*** **=** **0.021** ^ǂ^(n.s.)
Kujala	Not performed	Not performed	78.0 (40–100)	Not applicable

* Pre-operative to 2-year FU; ^†^ Pre-operative to final FU; ^ǂ^ 2-year FU to final FU (all repeated-measures ANOVA test)

Post-operatively, a subjective feeling of patellar instability was reported continuously or at light exertion in six knees (five patients), from which objective patellar dislocations were reported in four knees (four patients; Table 2). This rate of patellar instability is higher than reported at the 2-year follow-up, at which time only two patellar dislocations had occurred, both within the first post-operative months. No knees showed swelling or effusion on physical examination. All knees had a full range of motion. A positive apprehension test for patellar dislocation was present in three of 14 knees, a positive J-sign in seven of 14 and the Rabot sign was positive in 12 of 14 knees (Table 3).

**Table 2:** The assessment of outcome for patellar instability

Patellar stability	*n* (%)
Catching of the patella	
Never	13 (86.7)
Sometimes	1 (6.7)
Often	1 (6.7)
Symptoms of patellar instability	
Never	8 (53.3)
With severe exertion	1 (6.7)
With light exertion	5 (33.3)
Continuous	1 (6.7)
Patellar dislocations	
None	11 (73.3)
Once	0 (0)
2–5 times	1 (6.7)
Over 5 times	3 (20)
Total	15 (100)

**Table 3:** Physical examination: patellofemoral evaluation (*n* = 14)

	*n*/*N* (%)
Positive Signe du Rabot	12/14 (86)
J-Sign present	7/14 (50)
Positive apprehension test	3/14 (21)

Patellofemoral evaluation in 14 knees, as one knee was not available for physical examination

**Fig. 2:**
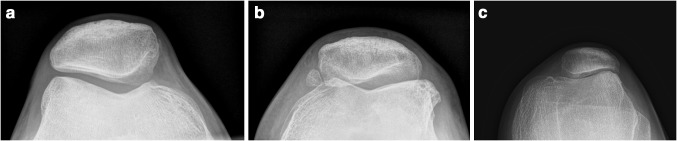
Conventional radiographs of the three grades of patellofemoral OA according to the Iwano classification showing **a** grade 0, no features of OA; **b** grade I, remodelling; **c** grade II, joint space narrowing of <3 mm

Radiological evaluation of osteoarthritis in the patellofemoral compartment according to the Iwano classification [12] (Fig. 2) is shown in Table 4. According to the K–L scale [14], assessment of osteoarthritis in the medial tibiofemoral compartment showed no osteoarthritis in seven of 15 knees (46.7%) and grade 1 in 8 of 15 knees (53.4%). On the lateral side, no osteoarthritis was seen in seven, grade 1 in seven and grade 2 in one of 15 knees (46.7, 46.7, and 6.7%, respectively). We observed an increase in radiological osteoarthritis in all three compartments when compared to pre-operative values; however, this was limited to the lower grades using both the Iwano and K–L classifications. In addition to these 15 knees, one patient (one knee) was converted to a patellofemoral arthroplasty due to progressive osteoarthritis and pain 3 years after her trochlear osteotomy. No correlation between VAS pain, Lysholm, WOMAC or Kujala scores and either patellofemoral or tibiofemoral osteoarthritis could be identified (n.s.).

**Table 4:** Radiological evaluation of patellofemoral and tibiofemoral osteoarthritis

Location	Classification	Pre-operative No of knees (%)	2-year FU No of knees (%)	12-year FU No of knees (%)
	Iwano			
Patellofemoral	None	14 (93.3)	13 (86.6)	3 (20)
	Stage 1	1 (6.7)	1 (6.7)	8 (53.3)
	Stage 2	0 (0)	1 (6.7)	4 (26.7)
Total		15 (100)	15 (100)	15 (100)

In addition to these 15 knees, one patient underwent a patellofemoral arthroplasty 3-year post-trochlear osteotomy

## Discussion

The most relevant finding in the present study is that the clinical results of this lateral condyle-elevating osteotomy do not significantly deteriorate over time. Patellofemoral osteoarthritis was generally confined to the lower grades on the Iwano scale. However, a high percentage of patients reported residual patellofemoral instability, with objective signs and subjective symptoms of instability requiring additional surgery. We conclude that this procedure has acceptable clinical results and does not lead to rapidly progressive osteoarthritis. It therefore has a place in the treatment arsenal for patellofemoral instability; however, it should not be performed as a stand-alone procedure since it is insufficient in guaranteeing patellar stability.

This is the largest study to date reporting the results of this type of osteotomy and contains the longest follow-up period. Only two previous studies of poor methodologic quality have been published on the results of this type of osteotomy. In 2003, Badhe et al. [3] reported on four patients with a follow-up of just 1 year. They performed a lateral condyle-elevating trochleoplasty in combination with an osteotomy of the patella and a tibial tuberosity transfer. No instability was found; however, all patients had a loss of flexion and residual patellofemoral pain. In 1997, Weiker and Black [34] reviewed five patients (six knees) with an average follow-up of 7 years. All patients had a deficient lateral condyle, and 3.8 previous surgical procedures on average, including two complete patellectomies. They found persistent symptoms in all six knees studied: four during sport activities, one during activities of daily living, and one knee was eventually converted to an arthrodesis. In two of four knees in which radiographic follow-up was available, osteophytes were present. When compared to our study, these results appear inferior both clinically and radiographically.

The VAS pain, Lysholm and WOMAC scores in our study all showed improvement from the pre-operative measurement to the final evaluation, and the median Kujala score at final follow-up was 76. Most authors describe the results at short- or mid-term follow-up. In a recent systemic review by Balcarek et al. [4], the results of a sulcus-deepening trochleoplasty were reviewed. They included six studies with a total of 186 knees, with and without concomitant stabilizing procedures. The mean follow-up was 44 months, and four of six studies had a follow-up period of less than 3 years. The mean post-operative Kujala score ranged from 81.7 to 92.1, and the mean increase in Kujala score was 26.2 points. Few authors have published results after a longer follow-up period [6, 22, 33]. Dejour et al. [6] reported a Kujala score of 81 at a mean follow-up of 66 months, and Ntagiopoulos et al. [22] published a Kujala score of 87 at a mean of 7-year follow-up, both after a Dejour-type trochleoplasty. Both these studies were incorporated into the systematic review by Balcarek et al. [4]. Von Knoch et al. [33] reported a mean post-operative Kujala score of 95 in 45 knees after a Bereiter trochleoplasty at 8-year follow-up. In these series, the authors described highly variable results: patellofemoral pain post-operatively improved in 22 of 45 knees, but worsened in 15 of 45 knees. Unfortunately, we are unable to report a difference in pre- and post-operative Kujala scores, as this was not pre-operatively recorded; however, it is clear that the mean Kujala scores reported in other studies are higher than those seen in our results.

The reported recurrence of patellar instability in our study (Table 3) is higher than that reported in other studies. Beaufils et al. [5] published an overview of the incidence of recurrent dislocations after trochlear osteotomies and reported a maximum of 10% (two of 20 patients) persistent objective patellar instability in a study by Thaunat et al. [30] and 2% in two other studies [10, 23]. No persistent instability was reported in the other six studies [9, 11, 20, 31–33] included in their review. These results were confirmed by Balcarek et al. [4], who found recurrent instability in four of 186 knees (2.1%) in their systematic review. However, most patients in these studies underwent additional procedures during the index procedure, such as an MPFL reconstruction, lateral release, medial reefing or tibial tuberosity transfers. In our study, no additional procedures were performed during the index surgery. Additional realignment procedures were performed in seven of 15 knees prior to surgery and in three knees at a later stage when there was persistent patellar instability. At the latest follow-up, six patients reported symptoms of patellar instability in seven knees, from which four patients reported recurrent patella dislocations in four knees (Table 2). Given the results of our study and those mentioned above, we recommend that this trochlear osteotomy is not performed as a stand-alone procedure, but that it is combined with a medial soft tissue stabilizing technique such as an MPFL reconstruction.

Pre-operatively, we observed no osteoarthritis in 14 of 15 knees; at final follow-up, we observed radiological patellofemoral osteoarthritis Iwano grade 1 or 2 in 12 of 15 knees [12], and one patient underwent a patellofemoral arthroplasty. This is higher than the occurrence of osteoarthritis after conservative treatment for patellar instability, which has been reported to be 29% (6/21) classified by the Ahlbäck scale at 14-year follow-up [2]. From the few studies with a longer follow-up period, Von Knoch et al. [33] found a similar incidence of degenerative changes in the patellofemoral joint (73%, 24 of 33 knees) at 8-year follow-up after a Bereiter trochleoplasty. On the Iwano scale, they reported grade 1 osteoarthritis in 14 knees, grade 2 in seven, grade 3 in two and grade 4 in one knee. Ntagiopoulos et al. [22] remarkably found no radiological osteoarthritis at a mean of 7-year follow-up after their sulcus-deepening trochleoplasty. In contrast, Rouanet et al. [24] found grade 2 or higher radiological osteoarthritis using the Iwano scale in 20 of 34 knees at 15-year follow-up in their series of a sulcus-deepening trochleoplasty, and seven knees had been converted to a total knee arthroplasty after the same procedure.

General perception of the Albee or lateral condyle-elevating procedures is that by raising the lateral condyle, pressures in the patellofemoral joint increase and thus lead to patellofemoral osteoarthritis. This idea is supported by a study by Kuroda et al., who found increased contact pressures in a cadaver model after the lateral trochlea was raised [18]. In contrast, other authors have reported that a larger sulcus angle, i.e. a flatter trochlea, especially in the proximal trochlea, leads to increased cartilage loss [13, 27, 29]. Raising the lateral condyle, and thus restoring the normal inclination of the lateral wall of the trochlea, then acts to preserve the cartilage of the patellofemoral joint instead of leading to an increase in osteoarthritis. Based on our results and the results reported in other studies, it can be concluded that a lateral condyle-elevating trochlear osteotomy leads to an increase in patellofemoral osteoarthritis, but that it does not exceed the degree of osteoarthritis reported in other types of trochleoplasty.

The major strength of this study is its long-term follow-up period and thorough evaluation at the final follow-up with physical examination and radiographs. Due to the nature of this procedure, it is indicated in relatively few cases of patellar instability. In this study, only 19 knees were included in the original series, of which 15 were available for final follow-up. Therefore, four of 19 knees (21.1%, including one patient who could not be evaluated due to conversion to patellofemoral arthroplasty) were unavailable for follow-up, diminishing the power of the study. Additionally, Kujala scores were not pre-operatively collected, increasing the difficulty of comparing these results to those of other studies, as pre- and post-operative differences could not be determined.

## Conclusion

In this study, the development of patellofemoral osteoarthritis after 12-year follow-up was not found to exceed the findings from other trochleoplasty case series. However, it is here concluded that a single lateral condyle-elevating trochlear osteotomy leads to a high amount of residual patellofemoral instability when compared to that reported in studies in which trochleoplasty was combined with other realignment procedures. In our opinion, a lateral condyle-elevating trochlear osteotomy has a role in the treatment of patellar instability; however, we recommend not using it as a stand-alone procedure but rather performing a simultaneous medial patellofemoral ligament reconstruction.

## References

[1] Albee FH (1915). The bone graft wedge in the treatment of habitual dislocation of patella. Med Records.

[2] Arnbjornsson A, Egund N, Rydling O, Stockerup R, Ryd L (1992). The natural history of recurrent dislocation of the patella. Long-term results of conservative and operative treatment. J Bone Joint Surg Br.

[3] Badhe NP, Forster IW (2003). Patellar osteotomy and Albee’s procedure for dysplastic patellar instability. Eur J Orthop Surg Traumatol.

[4] Balcarek P, Rehn S, Howells NR, Eldridge JD, Kita K, Dejour D, Nelitz M, Banke IJ, Lambrecht D, Harden M, Friede T (2016). Results of medial patellofemoral ligament reconstruction compared with trochleoplasty plus individual extensor apparatus balancing in patellar instability caused by severe trochlear dysplasia: a systematic review and meta-analysis. Knee Surg Sports Traumatol Arthrosc.

[5] Beaufils P, Thaunat M, Pujol N, Scheffler S, Rossi R, Carmont M (2012). Trochleoplasty in major trochlear dysplasia: current concepts. Sports Med Arthrosc Rehabil Ther Technol.

[6] Dejour D, Byn P, Ntagiopoulos PG (2013). The Lyon’s sulcus-deepening trochleoplasty in previous unsuccessful patellofemoral surgery. Int Orthop.

[7] Dejour D, Saggin P (2010). The sulcus deepening trochleoplasty: the Lyon’s procedure. Int Orthop.

[8] Dejour H, Walch G, Nove-Josserand L, Guier C (1994). Factors of patellar instability: an anatomic radiographic study. Knee Surg Sports Traumatol Arthrosc.

[9] Donell ST, Joseph G, Hing CB, Marshall TJ (2006). Modified Dejour trochleoplasty for severe dysplasia: operative technique and early clinical results. Knee.

[10] Gougeon FVP, Migaud H, Debroucker MJ, Spiers A, Duquennoy A (1996). Résultats après 3 ans de recul de 51 trochléoplasties pour instabilité frontale fémoropatellaire. Rev Chir Orthop Reparatrice Appar Mot.

[11] Goutallier D, Raou D, Van Driessche S (2002). Retro-trochlear wedge reduction trochleoplasty for the treatment of painful patella syndrome with protruding trochleae. Technical note and early results. Rev Chir Orthop Reparatrice Appar Mot.

[12] Iwano T, Kurosawa H, Tokuyama H, Hoshikawa Y (1990). Roentgenographic and clinical findings of patellofemoral osteoarthrosis. With special reference to its relationship to femorotibial osteoarthrosis and etiologic factors. Clin Orthop Relat Res.

[13] Kalichman L, Zhang Y, Niu J, Goggins J, Gale D, Felson DT, Hunter D (2007). The association between patellar alignment and patellofemoral joint osteoarthritis features: an MRI study. Rheumatology (Oxford).

[14] Kellgren JH, Lawrence JS (1957). Radiological assessment of osteo-arthrosis. Ann Rheum Dis.

[15] Koeter S, Bongers EM, de Rooij J, van Kampen A (2006). Minimal rotation aberrations cause radiographic misdiagnosis of trochlear dysplasia. Knee Surg Sports Traumatol Arthrosc.

[16] Koeter S, Pakvis D, van Loon CJ, van Kampen A (2007). Trochlear osteotomy for patellar instability: satisfactory minimum 2-year results in patients with dysplasia of the trochlea. Knee Surg Sports Traumatol Arthrosc.

[17] Kujala UM, Jaakkola LH, Koskinen SK, Taimela S, Hurme M, Nelimarkka O (1993). Scoring of patellofemoral disorders. Arthroscopy.

[18] Kuroda R, Kambic H, Valdevit A, Andrish J (2002). Distribution of patellofemoral joint pressures after femoral trochlear osteotomy. Knee Surg Sports Traumatol Arthrosc.

[19] LaPrade RF, Cram TR, James EW, Rasmussen MT (2014). Trochlear dysplasia and the role of trochleoplasty. Clin Sports Med.

[20] Masse Y (1978). Trochleoplasty. Restoration of the intercondylar groove in subluxations and dislocations of the patella. Rev Chir Orthop Reparatrice Appar Mot.

[21] Ntagiopoulos PG, Dejour D (2014). Current concepts on trochleoplasty procedures for the surgical treatment of trochlear dysplasia. Knee Surg Sports Traumatol Arthrosc.

[22] Ntagiopoulos PG, Byn P, Dejour D (2013). Midterm results of comprehensive surgical reconstruction including sulcus-deepening trochleoplasty in recurrent patellar dislocations with high-grade trochlear dysplasia. Am J Sports Med.

[23] Reynaud P (1995) Les trochleoplasties -creusement. In: 8emes Journees Lyonnaises de chirurgie de genou. sauramps Med edition, Montpellier, pp 176–190

[24] Rouanet T, Gougeon F, Fayard JM, Remy F, Migaud H, Pasquier G (2015). Sulcus deepening trochleoplasty for patellofemoral instability: a series of 34 cases after 15 years postoperative follow-up. Orthop Traumatol Surg Res.

[25] Senavongse W, Amis AA (2005). The effects of articular, retinacular, or muscular deficiencies on patellofemoral joint stability: a biomechanical study in vitro. J Bone Joint Surg Br.

[26] Steensen RN, Bentley JC, Trinh TQ, Backes JR, Wiltfong RE (2015). The prevalence and combined prevalences of anatomic factors associated with recurrent patellar dislocation: a magnetic resonance imaging study. Am J Sports Med.

[27] Stefanik JJ, Roemer FW, Zumwalt AC, Zhu Y, Gross KD, Lynch JA, Frey-Law LA, Lewis CE, Guermazi A, Powers CM, Felson DT (2012). Association between measures of trochlear morphology and structural features of patellofemoral joint osteoarthritis on MRI: the MOST study. J Orthop Res.

[28] Tegner Y, Lysholm J (1985). Rating systems in the evaluation of knee ligament injuries. Clin Orthop Relat Res.

[29] Teichtahl AJ, Hanna F, Wluka AE, Urquhart DM, Wang Y, English DR, Giles GG, Cicuttini FM (2012). A flatter proximal trochlear groove is associated with patella cartilage loss. Med Sci Sports Exerc.

[30] Thaunat M, Bessiere C, Pujol N, Boisrenoult P, Beaufils P (2011). Recession wedge trochleoplasty as an additional procedure in the surgical treatment of patellar instability with major trochlear dysplasia: early results. Orthop Traumatol Surg Res.

[31] Utting MR, Mulford JS, Eldridge JD (2008). A prospective evaluation of trochleoplasty for the treatment of patellofemoral dislocation and instability. J Bone Joint Surg Br.

[32] Verdonk R, Jansegers E, Stuyts B (2005). Trochleoplasty in dysplastic knee trochlea. Knee Surg Sports Traumatol Arthrosc.

[33] von Knoch F, Bohm T, Burgi ML, von Knoch M, Bereiter H (2006). Trochleaplasty for recurrent patellar dislocation in association with trochlear dysplasia. A 4- to 14-year follow-up study. J Bone Joint Surg Br.

[34] Weiker GT, Black KP (1997). The anterior femoral osteotomy for patellofemoral instability. Am J Knee Surg.

